# Optimising the Use of TRIzol-extracted Proteins in Surface Enhanced Laser Desorption/ Ionization (SELDI) Analysis

**DOI:** 10.1186/1477-5956-4-3

**Published:** 2006-03-23

**Authors:** Tsz-Kwong Man, Yiting Li, Tu Anh Dang, Jianhe Shen, Laszlo Perlaky, Ching C Lau

**Affiliations:** 1 Texas Children's Cancer Center, Department of Pediatrics, Baylor College of Medicine, Houston, TX, USA; 2 Ciphergen Biosystems, Fremont, CA, USA

## Abstract

**Background:**

Research with clinical specimens is always hampered by the limited availability of relevant samples, necessitating the use of a single sample for multiple assays. TRIzol is a common reagent for RNA extraction, but DNA and protein fractions can also be used for other studies. However, little is known about using TRIzol-extracted proteins in proteomic research, partly because proteins extracted from TRIzol are very resistant to solubilization.

**Results:**

To facilitate the use of TRIzol-extracted proteins, we first compared the ability of four different common solubilizing reagents to solubilize the TRIzol-extracted proteins from an osteosarcoma cell line, U2-OS. Then we analyzed the solubilized proteins by Surface Enhanced Laser Desorption/ Ionization technique (SELDI). The results showed that solubilization of TRIzol-extracted proteins with 9.5 M Urea and 2% CHAPS ([3-[(3-cholamidopropyl)-dimethylammonio]propanesulfonate]) (UREA-CHAPS) was significantly better than the standard 1% SDS in terms of solubilization efficiency and the number of detectable ion peaks. Using three different types of SELDI arrays (CM10, H50, and IMAC-Cu), we demonstrated that peak detection with proteins solubilized by UREA-CHAPS was reproducible (r > 0.9). Further SELDI analysis indicated that the number of ion peaks detected in TRIzol-extracted proteins was comparable to a direct extraction method, suggesting many proteins still remain in the TRIzol protein fraction.

**Conclusion:**

Our results suggest that UREA-CHAPS performed very well in solubilizing TRIzol-extracted proteins for SELDI applications. Protein fractions left over after TRIzol RNA extraction could be a valuable but neglected source for proteomic or biochemical analysis when additional samples are not available.

## Background

TRIzol is a common RNA extraction reagent that has been extensively used in conjunction with microarray analysis and other applications [1-4]. One of the advantages of TRIzol is its capability of extracting RNA, DNA, and proteins from a single sample. However, TRIzol is primarily designed for RNA extraction and the use of TRIzol extracted DNA and proteins for subsequent analysis is still limited. DNA and protein fractions from TRIzol extraction are valuable resources for researchers when the quantity of the starting material is limited, such as small clinical specimens. In addition, if different fractions extracted from the same sample are used for analysis on various high-throughput platforms, such as expression array, SNP array, and proteomic analyses, correlation of the resultant data sets will be less likely to be affected by tissue heterogeneity [5,6]. We have reported that whole genome-amplified DNA extracted from TRIzol fraction revealed similar genotypic aberrations in Affymetrix 10 K SNP arrays when compared to unamplified DNA and traditional comparative genomic hybridization [7]. However, the usability of TRIzol-extracted proteins in proteomics applications, such as mass spectrometry-based technology is still largely unknown. One of the reasons is that TRIzol-extracted proteins are very resistant to solubilization using the standard solubilizing reagent, 1% SDS as recommend by the TRIzol user manual. This hampers the use of these proteins for subsequent analysis.

To address this problem, we have evaluated the solubilization efficiency of TRIzol-extracted proteins by four commonly used solubilizing reagents. We also examined the number of ion peaks detected and the reproducibility of peak intensity of the solubilized proteins on three different array types of Surface Enhanced Laser Desorption/ Ionization (SELDI). In addition, the combinations of solubilizing regent and array type for TRIzol-extracted proteins were evaluated. We chose SELDI to analyze the proteins because it is a rapid and sensitive high-throughput proteomic technique, which has been used to discover protein biomarkers in basic and clinical studies using limited amount of materials [8,9]. This is the first report of using TRIzol-extracted proteins in SELDI studies.

## Results and discussion

### Solubilization efficiency of various reagents

**Figure 1:**
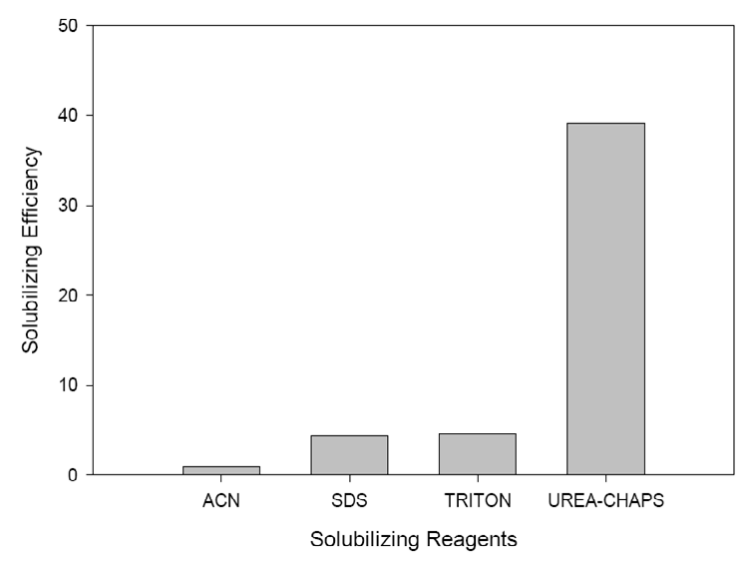
Extraction efficiency of the four solubilizing reagents.

To identify the best solubilizing reagent, TRIzol-extracted proteins of a human osteosarcoma cell line (U-2O S) were first solubilized with four solubilizing reagents, namely ACN (10% Acetonitrile, pH 4.8), TRITON (1% Triton, pH 5.3), UREA-CHAPS (9.5 M Urea and 2% CHAPS, pH 9.1) and SDS (1% SDS, pH 5.3). These four solubilizing reagents were chosen in this study because they are commonly used in dissolving various protein samples. The solubilization efficiency was calculated as the percentage of the amount of solubilized proteins divided by the weight of the initial protein pellet. The results showed that UREA-CHAPS solubilized significantly higher amount of proteins than the other three solubilizing reagents (Fig. 1). The solubilization efficiency of UREA-CHAPS was 8.8-fold higher than that of the standard 1% SDS, which is often used to dissolve TRIzol-extracted proteins. In addition to the intrinsic differences of the reagents, pH of the solvent also plays an important role in the solubilizing efficiency. Our finding is consistent with Banerjee et al [10], which showed that higher pH significantly increases the yield of the total protein extracted from TRIzol protein pellets.

### Number of ion peaks detected by SELDI

Although UREA-CHAPS gave higher solubilization efficiency, we were not sure if the solubilized mixture is suitable for proteomic analysis. Therefore, we used a proteomic technique called SELDI to measure the number of detectable proteins in the solubilized mixtures. Solubilized proteins from different experiments were spotted onto three different types of SELDI arrays, hydrophobic H4, weak cationic CM10, and metal (Copper) binding IMAC-Cu. These three different types of arrays have different chemical properties for binding to different subsets of proteins in the solubilized protein mixtures. Using different combinations of solubilizing reagents and array types, we could optimize the condition for analyzing the TRIzol-extracted proteins using SELDI method.

**Figure 2:**
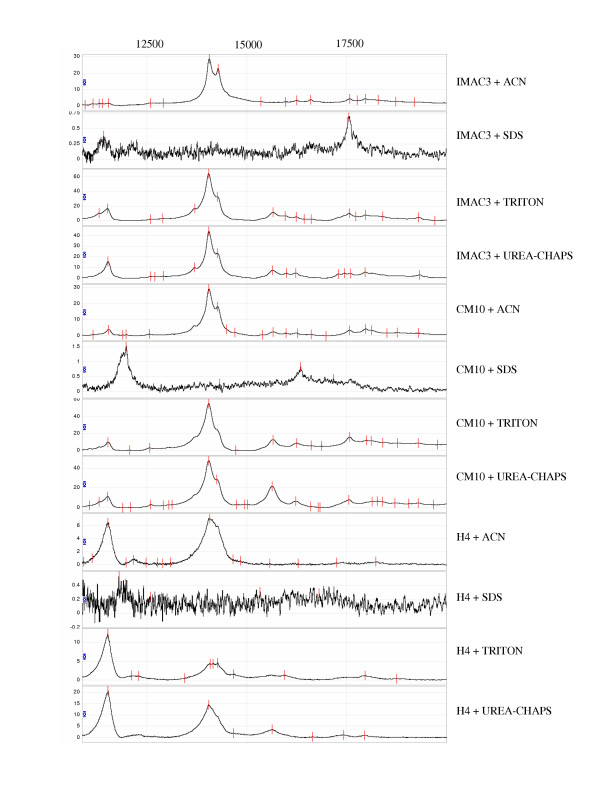
Representative SELDI spectra of four solubilizing reagents and three types of array at the high laser energy (see text for details). The x-axis represents the M/Z ratio and the y-axis represents intensity of the peaks. Peaks (tick marks) were detected by Ciphergen's Biomarker Wizard software (Ciphergen Biosystems, Fremont, CA).

**Figure 3:**
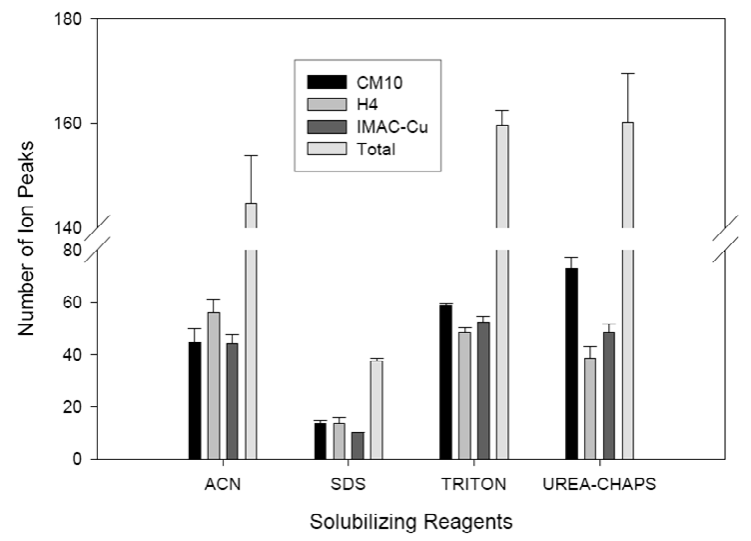
Number of peaks detected by SELDI experiments using various solubilizing reagents and array types. The y-axis values represent the mean values of the three replicates performed on different days.

In order to detect proteins with different molecular weights, two levels of laser energy (low and high) were used in the SELDI analysis. After the corresponding profiles were collected (Fig. 2), the number of detected ion peaks was measured. We found that the number of ion peaks increased with increase of laser energy (Table 1). This is consistent with the fact that we have a larger mass range in the higher laser setting when compared to the lower laser settings. Among the four solubilizing reagents, we detected the lowest number of ion peaks (in both low and high laser settings) in the standard 1% SDS solubilized protein preparation with all three-array types (peak number = 10 – 14, Fig. 3). However, when we combined all peaks detected by the three arrays, the highest number of ion peaks was detected in TRITON or UREA-CHAPS solubilized protein preparation (peak number is around 160, Fig 3). Although the three different arrays are designed to capture proteins with different chemical properties, some proteins may bind to multiple array types. Therefore the number of unique peaks might be less than the numbers shown above. Interestingly, the number of solubilized proteins detected by SELDI did not correlate with the protein concentrations of the protein mixtures, suggesting that the number of ion peaks detected by SELDI may be affected by other factors other than the protein concentration, such as binding efficiency of the solubilizing reagents to the arrays. Alternatively, the sensitivity of SELDI may be already high enough to detect ion peaks in the samples with low protein concentrations. When compared to various array types, the combination of UREA-CHAPS reagent and CM10 array captured the highest number of peaks (peak number = 75, Fig. 3). However, TRITON solubilized protein preparations showed a relatively more consistent number of detectable peaks in all three types of array (peak number = 48–58, Fig. 3).

**Table 1:** Comparison of the number of peaks detected in direct and TRIzol extractions using UREA-CHAPS reagent and CM10 array.

		Number of peaks detected by CM10				
		
Laser Power	M/Z Range (x1000)	Direct extraction	TRIzol extraction			
		
		Urea	Urea	Triton	ACN	SDS
Low	2 to 10	14	15	14	12	2
High	10 to 200	60	60	45	33	12
Total	2 to 200	74	75	59	45	14

### Reproducibility

**Figure 4:**
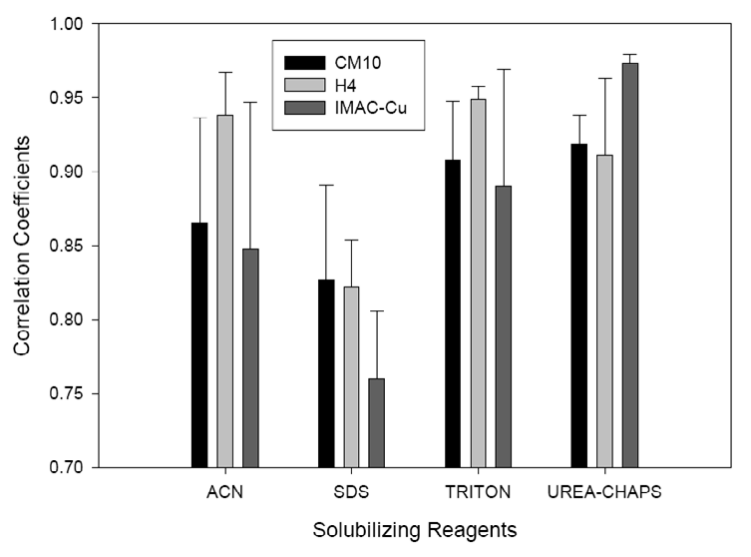
The reproducibility of peak intensities in SELDI experiments using various solubilizing reagents and array types. The y-axis values represent the mean values of the three replicates performed on different days.

Next, we measured the reproducibility of the solubilization and detection by calculating the pair-wise parametric Pearson's coefficients of the peak intensities in triplicate solubilization experiments performed on different days (Fig. 4). In addition, the reproducibility of the duplicated spots of the same experiment was also calculated. The Pearson's coefficients of CM10, H4, and IMAC-Cu were 0.96, 0.82, 0.90, respectively. The high correlation of the duplicate spots of the same experiment suggested that the variability of the SELDI measurements was low. The reproducibility result of CM10 is consistent with a recent study using the same chip type [11].

When compared to the overall reproducibility, the standard 1% SDS solubilization was the lowest among all four solubilization reagents tested (r = 0.76 – 0.83, Fig. 4). ACN showed high reproducibility with H4 array and TRITON showed high reproducibility with CM10 and H4 arrays. However, only UREA-CHAPS solubilization showed consistently high reproducibility in all three array types (r > 0.9) (Fig. 4). UREA-CHAPS coupled with IMAC-Cu array achieved the highest reproducibility among all solubilizing reagent-array type combinations (Fig. 4).

### Comparison between direct and TRIzol extractions

Although we were mainly interested in using TRIzol-extracted proteins in this study, to evaluate the usefulness of TRIzol-extracted proteins in SELDI applications, we also compared the number of extractable proteins retained in the TRIzol protein fraction to that of a direct extraction method. For this purpose, UREA-CHAPS, which showed the highest efficiency of solubilizing TRIzol-extracted proteins in this study, was used to directly extract proteins from the U2-OS cell pellet. Peak numbers detected by SELDI from the protein mixtures extracted from the two approaches were compared on CM10 arrays. (Table 1). The results showed that the number of peaks extracted from the direct method was very comparable to TRIzol extraction coupled with the same UREA-CHAPS solubilizing reagent (Table 1). The number of peaks detected in our study is also similar to a previous study using SELDI analysis on directly extracted proteins (peak number = 50). [12] Nevertheless, since proteins bound to a specific SELDI array is only a subset of cellular proteome, the actual number of protein species in the TRIzol-extracted proteins should be even higher. Furthermore, fractionation or immunodepletion will also further increase the number of proteins being detected by reducing the suppression effect of abundant proteins. Therefore, we believe that a significant fraction of proteins is still retained in TRIzol- extracted protein fractions. These protein fractions should be valuable for proteomic analysis, especially if another sample of the same tissue is not available.

## Conclusion

In this study, we evaluated the use of TRIzol-extracted proteins in SELDI-based proteomic analysis. Our study is useful for the clinical proteomics investigations, which may have only limited-sized samples and have to rely on TRIzol to extract both RNA and proteins from the same sample. Among the four solubilizing reagents tested, SDS-solubilized proteins showed the lowest peak numbers and poor reproducibility, suggesting the incompatibility of SDS with SELDI. Although SDS is recommend by the TRIzol manual, the use of SDS should be avoided in SELDI-based proteomic applications. On the other hand, UREA-CHAPS performed very well in solubilizing TRIzol-extracted protein. SELDI analysis detected large number of peaks in UREA-CHAPS solubilized, TRIzol-extracted proteins and showed consistent reproducibility among all three types of SELDI arrays used. It also solubilized similar number of peaks in TRIzol-extracted proteins when compared to the direct extraction method, suggesting that significant amount of proteins is still present in the TRIzol protein fraction. In addition, since UREA-CHAPS is a commonly used solubilizing or extraction reagent [13,14], the use of UREA-CHAPS to solubilize TRIzol-extracted proteins could be extended to other proteomic techniques, such as 2-D gel. From the results of our study, ACN, TRITON and UREA-CHAPS are all compatible with SELDI application. Since different chip types detect different subset of proteins, the selection of chip type should be based on the proteins of interest.

## Methods

### Reagents

TRIzol reagent was purchased from Invitrogen (Carlsbad, CA). Isopropanol, guanidine hydrochloride, [3-[(3-cholamidopropyl)-dimethylammonio]propanesulfonate] (CHAPS) were purchased from Sigma (St. Louis, MO). Acetonitrile and Urea were from Fluka (Buchs, Switzerland). Triton was from Supelco (Supelco Park, PA). McCoy's 5A medium, penicillin-streptomycin, trypsin-EDTA, phosphate-buffered saline and 10% SDS were from Gibco-Invitrogen (Grand Island, NY). Fetal bovine serum was fromGemini (Woodland, CA). DC protein assay Kit was from Bio-Rad (Hercules, CA).

### Cell culture

Human Osteosarcoma U-2 OS cell line (HTB-96) was purchased from American Type Culture Collection (Manassas, VA). Cells were cultured at 37°C in McCoy's 5A medium supplemented with 10% fetal bovine serum, 100 U/mL of penicillin and 100 μg/mL of streptomycin in a 5% CO_2 _environment.

### Extraction of Proteins using TRIzol

Cells were digested with 0.25% trypsin-EDTA, washed three times with phosphate-buffered saline and pelleted by centrifugation at 200 g for 10 min. The cell pellets were stored at -80°C prior to use. One milliliter of TRIzol reagent was added to every 60 mg of cell pellet. After removal of RNA and DNA, proteins were precipitated by isopropanol. The protein pellet was washed three times in 0.3 M guanidine hydrochloride/95% ethanol, then centrifuged and washed with 100% ethanol. After centrifugation and removal of ethanol, the pellet was dried in SpeedVac. Protein pellets were stored at -20°C prior to use.

### Dissolving protein pellet using solubilizing reagents

The starting protein pellet was first extracted from 30 mg of cell pellet. It was then divided into four portions and each portion was dissolved with 50 μl of one of the four solubilizing reagents for two hours: namely ACN (10% Acetonitrile, pH 4.8), TRITON (1% Triton, pH 5.3), UREA-CHAPS (9.5 M Urea and 2% CHAPS [3-[(3-cholamidopropyl)-dimethylammonio]propanesulfonate], pH 9.1) (ProteinChip Application Guide, Ciphergen Biosystems, Fremont, CA) and SDS (1% SDS, pH 5.3, TRIzol Reagent manual, Invitrogen, Carlsbad, CA). SDS solubilization was performed at 50°C with occasional vortex according to the TRIzol manual. Solubilization by other three reagents was performed at room temperature with shaking speed at 150 rpm (Ciphergen's ProteinChip Application Guide). After incubation, samples were centrifuged at 16,000 g for 10 min at 4°C to remove any particulate materials. Protein solutions were aliquoted and stored at -20°C prior to use. The solubilization process was repeated two more times at different days (n = 3) to measure the reproducibility of the solubilization methods. To compare the solubilization efficiency, protein concentrations of different solubilized samples were measured by Lowry-based DC (detergent-compatible) protein assay. For solubilization efficiency test, the protein pellets were weighed and the solubilization efficiency was calculated as the percentage of the amount of the solubilized proteins divided by the weight of the protein pellet.

### Capture of solubilized proteins using SELDI arrays

Equal volumes (5 μl) of solubilized proteins diluted with binding buffer (1:5) from different experiments were spotted onto three different types of SELDI arrays, hydrophobic H4, weak cationic CM10, and metal (Copper) binding IMAC3 (Ciphergen Biosystems, Fremont, CA). The pH of the diluted samples was confirmed to be the same before applying onto the array. Capture of proteins was performed according to the manufacturer's instruction (Ciphergen Biosystems, Fremont, CA). For H4 array binding, arrays were pretreated with 75% acetonitrile in a humidified chamber for 3 min. After rinsing with HPLC grade water, 5 μl of sample that has been diluted (1:5) in 5% acetonitrile was applied to each spot on the array. The arrays were incubated for 20 min and washed three times with PBS containing 10% Acetonitrile and 100 mM NaCl. After air drying, 0.5 μl of saturated alpha-cyano-4-hydroxy cinnamic acid (CHCA) was applied twice onto each spot.

For CM10 array binding, spots were pre-wetted twice with CM10 low stringency buffer (0.1 M sodium acetate, pH 4.0) for 5 min. Then 5 μl of sample diluted in low stringency buffer (1:5) was applied to each spot and incubated for 1 h. Spots were washed three times with low stringency buffer and then twice with HPLC grade water. After air drying, 0.5 μl of saturated Sinapinic acid (SPA) was applied twice onto each spot.

For IMAC3 array binding, spots were coated twice with charging solution (100 mM copper sulfate) for 15 min. Arrays were then rinsed with running HPLC grade water for 10 s to remove copper and then rinsed with neutralization solution (100 mM sodium acetate, pH 4). After rinsing with running water for another 10 s, spots were incubated in 5 μl of binding buffer (0.5 M NaCl in PBS) for 10 min. Five microliters of sample diluted in binding buffer (1:5) was applied to each spot and incubated for 1 h after which the spots were rinsed 6 times with binding buffer and then twice with HPLC grade water. After air drying, 0.5 μl of saturated SPA was applied onto each spot twice.

### SELDI and data analysis

Arrays were placed in the Protein Biological System II mass spectrometer reader (Ciphergen Biosystems, Fremont, CA) and time-of-flight spectra were generated using two different (low and high) laser spot protocols. The low laser spot protocol was performed to detect proteins of low molecular weight with settings of laser intensity 185, detector sensitivity 6 and optimization range from 1000 Da to 9000 Da. The high laser spot protocol with settings of laser intensity 250, detector sensitivity 8 and optimization range from 10 kDa to 150 kDa was applied to detect high molecular weight proteins. Center pulse and automatic deflector settings were applied in spot protocols.

Peak detection was performed using ProteinChip Software 3.1 (Ciphergen Biosystems, Fremont, CA). The molecular mass below 2000 Da were eliminated from analysis because this area contains adducts and artifacts of the Energy Absorbing Molecule (EAM) and possibly other chemical contaminants. Spectra of samples solubilized with the same reagent and generated under the same laser condition were grouped together and baseline subtracted. The spectra were normalized to the total ion current of m/z starting from 1500 for the low molecular weight protein or from 10,000 for the high molecular weight protein. Peaks were autodetected with a signal to noise ratio of >5 and the peaks were clustered using second pass peak selection with signal to noise ratio of >2. A 0.7% mass window was selected to obtain optimal label of peaks. Peak information was exported into Excel and peak number was calculated for each spectra. For the analysis of reproducibility, estimated peaks were added to make sure that the peak information is complete for each cluster.

SPSS statistical software (SPSS, Chicago, IL) was used for the calculation of Pearson's coefficients. In our reproducibility analysis, all the peaks were used for correlation calculations. For the spot duplicates, they were done on the same day, but the repeated experiments were done on different days. The data from duplicate spots were first averaged before they were used in the calculation of the reproducibility of results obtained from replicate experiments done on different days.

## Competing interests

The author(s) declare that they have no competing interests. TAD is an employee of Ciphergen Biosystems, Inc, the manufacturer of SELDI arrays and reader.

## Authors' contributions

Yiting Li and Jianhe Shen were involved in the technical execution of the project. Tu Anh Dang was involved in data analysis and execution of some of the experiments. Laszlo Perlaky prepared the cell culture. Ching Lau provided project direction and funding of this project, and participated in the preparation of the manuscript. Tsz-Kwong Man analysed the data and directed the study.
